# Challenges of ageing in prisons and forensic psychiatric settings

**DOI:** 10.1192/j.eurpsy.2022.1554

**Published:** 2022-09-01

**Authors:** C. Peixoto, D. Rego, M. Cruz, B. Peixoto, M. Bicho, J. Coelho, H. Medeiros

**Affiliations:** Hospital do Divino Espírito Santo de Ponta Delgada, Psychiatry, Ponta Delgada, Portugal

**Keywords:** Secure Services, forensic psychiatry, older offender, Ageing

## Abstract

**Introduction:**

There is a current trend towards an increase in the number of elderly prisoners due to the increase in life expectancy and the change in the attitude of society and the judicial system. The cut-off for “older offender” is defined from the age of 50, due to the lifestyle previous to prision and premature ageing.

**Objectives:**

The authors intend to understand the challenges of aging in prison and forensic services, highlighting the psychiatric comorbidities of inmates and how these services can adapt to the needs of this population.

**Methods:**

Non-systematic review of the literature.

**Results:**

Studies of elderly in prisons and elderly forensic psychiatric patients are limited. Prisoners have increased physical and psychiatric morbidity and early mortality as they are more exposed to risk factors and more likely to have at least one health problem compared to older adults in the community. Compared to older people in the community, older prisoners are at higher risk for most psychiatric disorders including depression, psychosis, bipolar disorder, cognitive impairment, personality disorder and anxiety. Suicide rates are also higher among elderly prisoners. The inadequacy of the prison system to respond to the unique needs of elderly prisoners has a detrimental impact on their overall experience of incarceration. The development of specific services for elderly prisoners or the adaptation of mixed units for the elderly population is proposed.

**Conclusions:**

The elderly population in prisons is growing and has higher risk of psychiatric pathology compared to community elders. Prison services with difficulties in identifying and meeting these needs.

**Disclosure:**

No significant relationships.

